# Urea Is Found in the Liver

**Published:** 1871-01

**Authors:** 


Urea is Found in the Liver.—The latest researches on the origin of urea have demonstrated that the kidneys do not secrete, but merely excrete urea, and that the liver is in part, at least, the source of it.—*Lancet*.

